# Detection of prespecified CE-associated pathogen targets by quantitative polymerase chain reaction is associated with CD138-positive endometrium: a retrospective cohort study

**DOI:** 10.3389/fimmu.2026.1928249

**Published:** 2026-10-13

**Authors:** Shogo Nishii, Nanako Iwami, Hirobumi Kamiya, Kiyotaka Kawai

**Affiliations:** 1 Department of Reproductive Medicine, Kameda IVF Clinic Makuhari, Chiba, Japan; 2 Department of Reproductive Medicine, Kamiya Ladies Clinic, Sapporo, Japan

**Keywords:** CD138, chronic endometritis, endometrial microbiota, non-*Lactobacillus*-dominant microbiota, qPCR

## Abstract

**Introduction:**

We examined whether endometrial quantitative polymerase chain reaction (qPCR) detection of DNA from prespecified pathogen targets classically implicated in chronic endometritis (CE), from other reproductive-tract bacterial targets, or from *Lactobacillus* is associated with CD138-defined CE, independent of incidental endometrial polyps.

**Methods:**

We included 126 infertile women who underwent concurrent CD138 immunostaining and endometrial qPCR and who had also undergone hysteroscopy. Women with antibiotic use within 1 month before sampling, *Lactobacillus* probiotic use, insufficient samples for CD138 assessment, or no hysteroscopy were excluded. Robustness was evaluated by two prespecified polyp-exclusion analyses, by exclusion of women with endometriosis or adenomyosis, and by multivariable logistic regression.

**Results:**

CD138-positive endometrium was detected in 57 of 126 women (45.2%). Detection of CE-associated pathogen targets was associated with CD138 positivity (odds ratio 8.01, 95% confidence interval 1.70–37.85, P = 0.003; exact conditional odds ratio 7.89, 1.61–76.56), with specificity 97.1%, positive predictive value 84.6%, and sensitivity 19.3%, but this rested on only 13 target-positive women, of whom 11 were CD138-positive, so the estimate is imprecise. Other reproductive-tract bacterial targets were not associated with CD138 positivity (odds ratio 0.55, 0.25–1.22, P = 0.171). *Lactobacillus* detection was not significantly associated with CD138 positivity (odds ratio 0.54, 0.27–1.11, P = 0.107), although several analyses were directionally consistent with lower reported *Lactobacillus* values in CD138-positive women; this was suggestive rather than confirmatory. Conversely, 80.7% (46/57) of CD138-positive women had no detectable pathogen target. After polyp exclusion, the association was maintained (odds ratios 17.44 and 18.79), whereas the other categories remained nonsignificant. The association persisted after exclusion of women with endometriosis or adenomyosis (odds ratio 8.32, 1.75–39.60) and in the parsimonious multivariable model (adjusted odds ratio 8.94, 1.85–43.24; Firth penalized adjusted odds ratio 7.40, 1.67–32.90).

**Discussion:**

Detection of prespecified CE-associated pathogen targets, but not detection of other reproductive-tract bacterial targets, was associated with CD138-positive CE, independent of incidental polyps; the *Lactobacillus* findings were suggestive but not confirmatory. Because the panel provides no universal bacterial quantification, these data cannot evaluate non-*Lactobacillus*-dominant microbiota as a community state. qPCR may help identify a high-specificity, DNA-defined pathogen-associated subset, while CD138-positive CE appears etiologically heterogeneous and still requires histologic diagnosis.

## Introduction

Chronic endometritis (CE) is a persistent, localized inflammation histologically characterized by CD138/syndecan-1-positive plasma cells in the endometrial stroma and has been implicated in infertility, recurrent implantation failure (RIF), and recurrent pregnancy loss (RPL) (1–5). Hematoxylin–eosin staining alone has low sensitivity because plasma cells are sparse and resemble stromal fibroblasts and mononuclear cells, whereas CD138 immunohistochemistry (IHC) improves detection and diagnostic reproducibility (6–8). McQueen et al. reported that CD138 IHC increased the apparent prevalence of CE in women with RPL from 13% to 56% and listed *Chlamydia trachomatis*, *Neisseria gonorrhoeae*, *Mycoplasma* and *Ureaplasma species*, *Escherichia coli*, and *Streptococcus*, *Staphylococcus*, and *Enterococcus* species as pathogens classically implicated in CE (1, 6, 9). However, diagnostic thresholds for CD138-positive CE remain heterogeneous across studies (7, 8).

The endometrium is no longer regarded as a sterile compartment. Culture-independent techniques have shown that the upper genital tract harbors a low-biomass but reproducible bacterial community whose composition differs along the vagina-cervix-uterus axis and varies with host factors such as menopausal status, body mass index, and vaginal pH; the *Lactobacillus*-depleted, anaerobe-enriched profiles that characterize vaginal dysbiosis have been linked not only to infertility but also to chronic inflammatory and proliferative endometrial conditions, supporting a model in which persistent dysbiosis maintains a pro-inflammatory endometrial microenvironment (10). Against this background, the diagnosis of CE is itself in transition. CD138 immunohistochemistry remains the operational standard but is limited by interobserver variability, the absence of a universally accepted cut-off, and false positivity arising from epithelial CD138 expression, and hysteroscopy, alternative plasma-cell markers such as MUM1, targeted quantitative polymerase chain reaction (qPCR), 16S ribosomal RNA sequencing, and uterine-fluid cytokine profiling are all being evaluated as complementary diagnostic vectors (11). The practical question is therefore not whether the endometrium harbors bacteria, but which molecular signal, a specific pathogen or a globally dysbiotic community, corresponds to the histologic lesion that is used to justify antimicrobial treatment.

Next-generation sequencing (NGS) studies have proposed non-*Lactobacillus*-dominant microbiota (NLDM), a *Lactobacillus*-depleted profile associated with poor reproductive outcomes (12–14). In clinical practice, commercialized qPCR panels are widely used for endometrial microbiological evaluation. Unlike NGS, which reports relative abundance and therefore mathematically links *Lactobacillus* depletion with increases in other fractions, qPCR provides absolute assessment of individual bacterial targets (12, 13, 15). Thus, qPCR permits target-specific assessment of CE-associated pathogen targets, of bacterial targets commonly found in NLDM profiles, and of *Lactobacillus* as independent variables against a histologic reference. Without universal bacterial quantification, however, it cannot reconstruct relative community composition or identify NLDM as a community state, and the question it can address is therefore whether CD138-defined inflammation is linked to detection of specific bacterial targets.

When CE is identified and treated with appropriate antibiotics, reproductive outcomes may approach those of unaffected women, and plasma cell resolution may be associated with improved pregnancy rates (9, 16–18). However, this study did not assess treatment response or reproductive outcomes. Conversely, indiscriminate antibiotic or probiotic treatment based solely on microbiota composition, without a histologic target, may cause unnecessary exposure, cost, and microbiota disturbance; whether CE should be screened before assisted reproductive technology remains debated (19).

Single and multiple endometrial polyps are a known cause of localized plasma cell infiltration and microbiota-independent CD138 positivity; together with micropolyps, they are often identified incidentally during hysteroscopy despite not being suspected on transvaginal ultrasound. A systematic review and meta-analysis reported a higher prevalence of CE in women with endometrial polyps than in women with a non-polypoid endometrium (pooled OR 3.07) (20). However, the relationship with CE may differ by polyp type: single/multiple polyps are estrogen-sensitive anatomical lesions and competing causes, whereas micropolyps are classically regarded as findings accompanying CE. If incidental polyps are unevenly distributed across qPCR categories, they could distort microbiota-CD138 associations. Therefore, we clarified which qPCR categories are associated with CD138-defined CE in a cohort that also underwent hysteroscopy and tested whether that association was independent of incidental polyps. The first objective was to determine whether prespecified pathogens classically implicated in CE are associated with CD138-defined CE. The second was to assess whether other reproductive-tract bacterial targets or *Lactobacillus* are associated with CD138 positivity and robust to polyp exclusion.

## Materials and methods

### Study design and participants

This single-center, retrospective cohort study was conducted at Kameda IVF Clinic Makuhari. The source population comprised women who were evaluated for infertility at our clinic between 1 May 2025 and 25 April 2026 and who had undergone CD138 immunostaining and endometrial qPCR concurrently as part of clinical care; this broadly defined infertile population included women with concurrent RPL or RIF as well as women with neither. Infertility was defined as failure to conceive after at least 12 months of regular unprotected intercourse in accordance with the definitions of the Japan Society of Obstetrics and Gynecology and the WHO; RPL was defined as two or more spontaneous miscarriages after ultrasound confirmation of an intrauterine pregnancy (chemical pregnancies were not included); and RIF was defined as two or more failed blastocyst transfers. The presence or absence of concurrent RPL and RIF was recorded. A subset of these women also contributed to a separate study from our group on vaginal Gram-stain morphotype scoring and endometrial pathogen detection, which used different eligibility criteria and did not include CD138 immunohistochemistry.

Reporting follows the STROBE statement, the EQUATOR Network reporting guideline for observational research. The recruitment process, from the source population through sequential exclusions to the analysis cohort and the sensitivity analyses, is shown in **Figure 1**.

**Figure 1:**
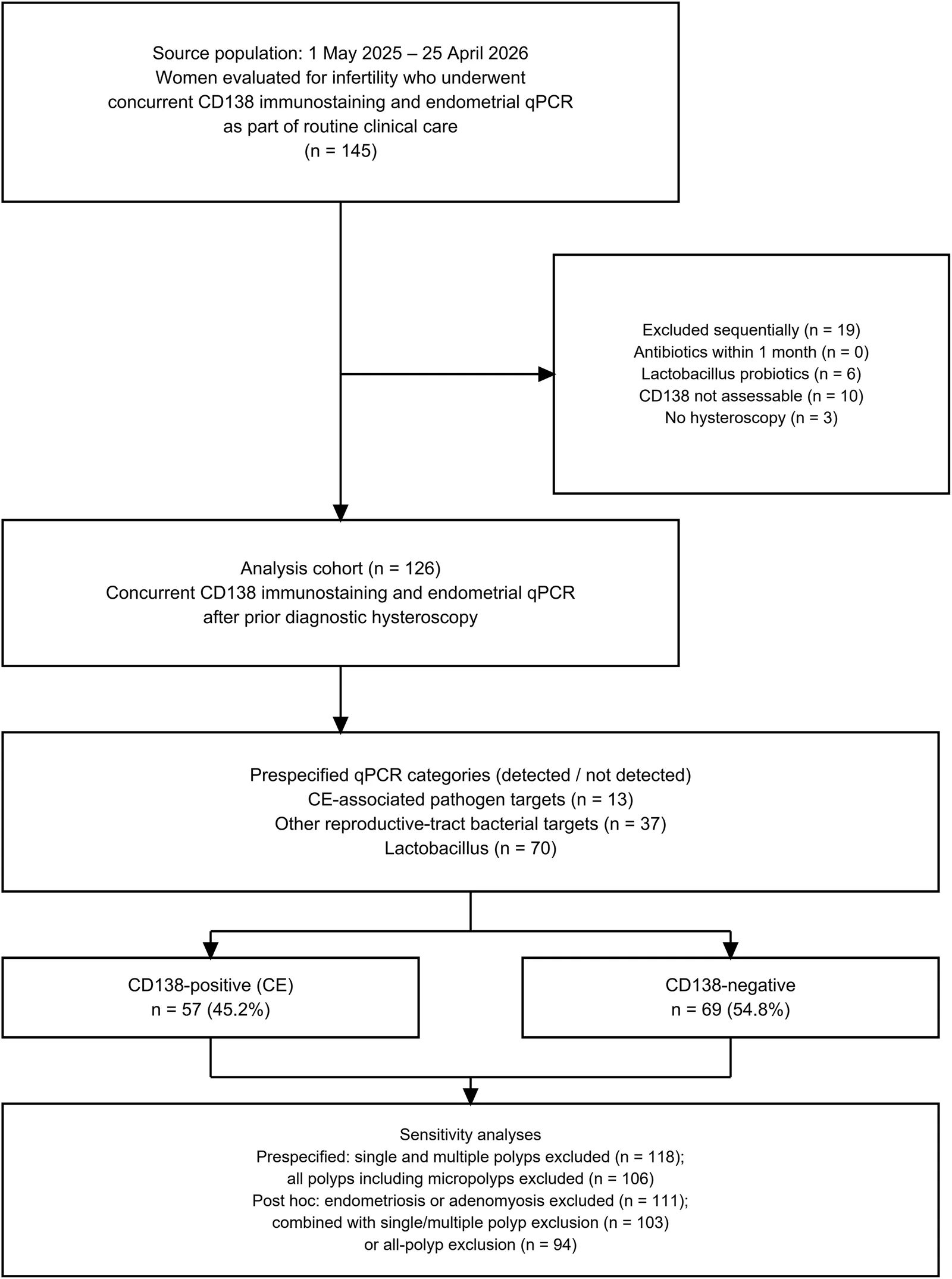
Recruitment and study flow (STROBE flow diagram). Of 145 women in the source population, 19 were excluded sequentially: none for antibiotic use within 1 month before sampling, 6 for *Lactobacillus* probiotic use, 10 because CD138 could not be assessed, and 3 because hysteroscopy was not performed. The analysis cohort comprised 126 women, of whom 57 (45.2%) were CD138-positive. The prespecified sensitivity analyses excluded single and multiple polyps (n = 118) and all polyps including micropolyps (n = 106). The post hoc sensitivity analyses excluded women with endometriosis or adenomyosis (n = 111), and these women together with single and multiple polyps (n = 103) or with all polyps (n = 94).

The indication for testing was broad: the combined evaluation was offered to women with RIF or RPL, and to women requesting endometrial evaluation before transfer. Accordingly, 67 of 126 women (53.2%) had neither RPL nor RIF (**Supplementary Table S1**). Because all women had to accept hysteroscopy, those with a longer treatment history may be over-represented.

The following women were sequentially excluded: (1) those who had received antibiotics within 1 month before sampling; (2) interview-confirmed users of oral or vaginal *Lactobacillus* probiotics (regardless of route, because exogenous *Lactobacillus* can alter both quantification and compositional classification); (3) those in whom CD138 could not be assessed because of insufficient sample volume; and (4) those who did not undergo hysteroscopy (hysteroscopy was required to evaluate polyps as a competing cause of CD138 positivity). The exclusion flow and the analysis cohort are shown in **Figure 1**. As background factors, adenomyosis and endometriosis were recorded based on transvaginal ultrasound and clinical evaluation at the start of fertility treatment (**Table 1**) but were not used as exclusion criteria. Because the immunostaining was performed as a clinical test and this study retrospectively analyzed already-obtained, anonymized clinical results without adding any new invasive procedures, an opt-out format was approved by the institutional ethics committee (approval number: WFC26-001).

**Table 1:** Background factors by CD138 status.

Characteristic	CD138-positive (n = 57)	CD138-negative (n = 69)	P value
Age (years), mean ± SD	36.5 ± 4.0	37.6 ± 4.2	0.139
BMI (kg/m²), mean ± SD	22.3 ± 3.8	21.8 ± 3.5	0.417
Recurrent pregnancy loss (≥2), n (%)	8 (14.0)	14 (20.3)	0.480
Recurrent implantation failure (≥2), n (%)	20 (35.1)	23 (33.3)	0.852
Adenomyosis, n (%)	1 (1.8)	4 (5.8)	0.377
Submucosal uterine fibroid, n (%)	0 (0.0)	0 (0.0)	– (NA)
Endometriosis, n (%)	7 (12.3)	5 (7.2)	0.375
Incidental polyp, n (%)	14 (24.6)	6 (8.7)	0.026
CE-associated pathogen targets, n (%)	11 (19.3)	2 (2.9)	0.003
Other reproductive-tract bacterial targets, n (%)	13 (22.8)	24 (34.8)	0.171
*Lactobacillus* detected, n (%)	27 (47.4)	43 (62.3)	0.107
Sampling timing: early secretory (POD 1–5), n (%)	40 (70.2)	52 (75.4)	0.47
mid-secretory (POD 6–8), n (%)	6 (10.5)	9 (13.0)	
late secretory (POD 9–13), n (%)	11 (19.3)	8 (11.6)	

All samples were obtained in the secretory phase, with no significant difference in CD138 positivity among subgroups (P = 0.47, chi-square test). Detection denotes qPCR amplification of target DNA at a Crt of 28 or less (the platform detection cut-off) and does not establish bacterial viability or active infection. The second category represents detection of individual bacterial targets that the manufacturer classifies as potentially pathogenic bacteria not related to chronic endometritis; it is not a measure of non-*Lactobacillus*-dominant microbiota. Abbreviations: CD138, syndecan-1; BMI, body mass index; SD, standard deviation; RPL, recurrent pregnancy loss; RIF, recurrent implantation failure; CE, chronic endometritis; POD, postovulatory day; NA, not applicable.

Women with recent antibiotic or probiotic exposure were excluded rather than adjusted for, because both alter the qPCR result itself: antibiotics remove the bacterial targets of the panel, and exogenous *Lactobacillus* cannot be distinguished from endogenous colonization. Since qPCR does not distinguish DNA from viable and non-viable bacteria, women who had received antibiotics within one month before sampling were excluded; in the event no woman in the source population had received antibiotics within that period (**Figure 1**), whereas six women were excluded for probiotic use.

### Histologic evaluation (CD138 and H&E)

Endometrial biopsies were obtained in the luteal phase and processed for H&E staining and CD138 IHC. Details of the biopsy procedure are provided in “Endometrial microbiota analysis.” CD138 immunostaining used a mouse monoclonal anti-human CD138 antibody (clone MI15; catalog no. M7228; Agilent Technologies) and was performed according to the standard immunostaining protocol of our institution’s pathology department. Plasma cell density was graded per high-power field, defined as ×400 total magnification. The number of HPFs examined was not fixed in advance; after the entire slide was examined, the most inflamed area was identified and scored in HPFs (equivalent to ×400) of that area. CE was defined as a CD138 score of ≥1 (McQueen grade 1, 2, or 3). H&E staining was performed and evaluated concurrently with CD138 IHC. Because CD138 IHC is significantly more sensitive than H&E staining for identifying plasma cells (6, 7), CD138 positivity was used as the histologic reference definition of CE in this study. Histologic evaluation was performed as an in-house clinical test independent of the qPCR performed by an external laboratory. As part of routine clinical double reading, two experienced pathologists reviewed the slides, focusing on CD138 staining and without reference to the qPCR results; discordant assessments, when present, were resolved by consensus. Formal interobserver agreement statistics (e.g., kappa) were not available because of the retrospective design. Architectural changes on H&E staining (e.g., stromal edema and spindling) were also observed concurrently; however, because substantial interobserver subjectivity has been reported (8), their systematic, quantitative scoring was not included as a requirement for the diagnosis of CE in this study.

The grading applied here follows the semiquantitative system of McQueen et al. (1, 6). After the whole section was screened, the most densely infiltrated (hotspot) area was identified and CD138-positive plasma cells were counted in high-power fields at ×400 total magnification and graded as follows: grade 0, none; grade 1, 1 to 5 plasma cells per HPF; grade 2, 6 to 20 per HPF; and grade 3, more than 20 per HPF or the presence of plasma-cell clusters. CE was defined as grade 1 or higher.

### Endometrial microbiota analysis

Endometrial biopsies were obtained in the luteal phase using a dedicated endometrial suction pipette (Pipelle-type catheter) through a standard technique that minimizes contamination from the lower genital tract. Endometrial sampling was performed as part of routine clinical care rather than according to a study protocol. Two reference points were used: the day of oocyte retrieval (i.e., the day of ovulation), and day 5 after the start of progesterone administration (P + 5) in ovulatory or hormone-replacement cycles. Because appointments were arranged around each woman’s treatment schedule, the interval between the reference point and the actual sampling date varied, and all samples were obtained during the secretory phase before the onset of luteal bleeding. Because the primary outcome was a binary determination of detection/nondetection rather than precise comparison of bacterial load, the effect of differences in sampling timing on the results is considered limited. After a sterile speculum was placed, the vagina and ectocervix were washed with saline, and cervical mucus was wiped off with a dry cotton ball. During catheter insertion, contact with the vaginal wall was avoided. Tissue was aspirated from the uterine cavity. As specified by the manufacturer, any potential microbial or cellular contamination originating from the cervix remains confined to the exterior surface of the catheter as it passes from the cervix to the uterus; consequently, during suction and scratching from the uterine fundus, only the endometrial lining tissue is drawn into the interior of the device, preserving the integrity of the collected sample (manufacturer’s operation manual). When the sample is transferred to the cryotube, the pipelle is not immersed in the preservative, so that only tissue retained inside the device enters the solution. Cervicovaginal contamination of endometrial samples can be minimized but not completely eliminated (a limitation common to all endometrial sampling); although the same sampling procedure was used in all women, contamination could not be excluded and its direction or magnitude could not be determined. In the analysis cohort, women who consumed antibiotics within 1 month before sampling were excluded; earlier antibiotic exposure before referral or off-site in several months before sampling could not be fully ascertained. Extracted DNA was analyzed using a customized semiquantitative qPCR panel targeting 26 potential pathogens and *Lactobacillus* (OpenArray platform, Thermo Fisher Scientific, Waltham, MA, USA). Species-specific TaqMan assays were used, with amplification and detection performed according to the manufacturer’s procedures. The panel is a commercially pre-designed panel and was used in its entirety, with no organism added or omitted by the investigators; in Japan, endometrial microbiota testing is available only through the assays approved under the Advanced Medical Care A framework, so the composition of the panel was not an investigator choice. Analysis was performed at an ISO-accredited, CLIA-certified central laboratory (Valencia, Spain). According to the manufacturer’s method performance specifications for this panel (Igenomix internal data), analytical sensitivity and specificity across all assays and controls were 99.63% and 99.83%, accuracy against contrived clinical samples was 99.30%, the between-run coefficient of variation was 3.4% (range 2.2–6.0%) and the within-run coefficient of variation 2.3% (range 0.5–8.1%), the linear dynamic range spanned 4 logs with R² > 0.97 for all assays, and the empirical limit of detection for most assays was 5–50 genomic copies per pre-amplification reaction (200–2,000 genomic copies per extraction). These are panel-level values. Target-specific limits of detection published by the manufacturer for the laboratory serving Europe, Asia, Africa and Oceania, expressed as the lowest spike-in dilution point detected, ranged from 0.3 to 400 genomic copies/µL (29) and are listed in **Supplementary Table S3**; no limit of quantification is specified. According to the manufacturer’s documentation (Informed Consent Form) for this test, after DNA is extracted from the endometrial tissue and before it is submitted for screening, each sample is assessed against minimum quality requirements; a sample with not enough human tissue is classified as “invalid” (verified using an internal control based on a specific human gene that must amplify within a validated threshold) and does not yield a result, requiring re-biopsy (i.e., reported results meet defined quality criteria). Each assay also includes internal controls that monitor contamination and technical performance. For each target the assay returns genome copies per mL (GC/mL), referred to 500 µL of a homogenate prepared from a piece of approximately 20 mg of the preserved biopsy in 600 µL of extraction buffer; according to the manufacturer, the usual tissue range of 10 to 40 mg corresponds to a variation of about ±1 Crt cycle. Ct/Crt values are retained by the laboratory and are not returned with the clinical report. Quantitative values for all targets are reported in **Supplementary Table S3**. Appropriate negative controls were incorporated throughout the analytical workflow to monitor for contamination. Because amplicon sequencing of low-biomass samples such as the endometrium can be affected by background contamination, targeted qPCR focused on clinically important species was chosen (21, 22). The reference ranges applied by the laboratory were established by the manufacturer from the analysis of Moreno et al. (13), being derived from endometrial biopsies of women who achieved a live birth in that multicenter prospective study; the detailed derivation has not been published separately. In all cases, endometrial samples were obtained in the secretory (postovulatory) phase. Based on the postovulatory day (POD), samples were classified into three groups: early secretory (POD 1–5, with the day of oocyte retrieval [day of ovulation] taken as POD 1; n = 92), mid-secretory (POD 6–8; n = 15), and late secretory (POD 9–13; n = 19).

For analysis, samples were classified into three prespecified categories based on detection (detected/not detected). The first category was qPCR detection of DNA from prespecified pathogen targets classically implicated in CE (hereafter “CE-associated pathogen targets”): *Chlamydia trachomatis*, *Enterococcus faecalis*, *Escherichia coli*, *Klebsiella pneumoniae*, *Mycoplasma genitalium*, *Mycoplasma hominis*, *Neisseria gonorrhoeae*, *Staphylococcus aureus*, *Streptococcus agalactiae* (group B)/viridans, and *Ureaplasma urealyticum*. The second category was the remaining 16 targets, which the manufacturer classifies as potentially pathogenic bacteria not related to chronic endometritis and which therefore fall neither within *Lactobacillus* nor within the manufacturer’s CE-associated group (hereafter “other reproductive-tract bacterial targets”): *Actinomyces israelii*, *Atopobium vaginae*, *Bacteroides fragilis*, *Bifidobacterium* spp., *Clostridium sordellii*, *Fusobacterium nucleatum*, *Gardnerella vaginalis*, *Haemophilus ducreyi*, *Mobiluncus* spp., *Mycobacterium tuberculosis*, *Peptostreptococcus anaerobius*, *Porphyromonas asaccharolytica*, *Prevotella bivia*, *Prevotella disiens*, *Sneathia* spp., and *Treponema pallidum*. The third category was detection of the genus *Lactobacillus*; the assay additionally reports four *Lactobacillus* species, which are analyzed separately in **Supplementary Table S5** and **Supplementary Figure S3**. *Lactobacillus* “status” in this study refers to the binary detection of the genus *Lactobacillus* and does not denote relative dominance or depletion. A target was classified as detected when amplification occurred at a relative threshold cycle (Crt) of 28 or less, the cut-off recommended by the manufacturer of the OpenArray platform as the minimum detectable value (corresponding to one copy), irrespective of whether the quantitative value exceeded the manufacturer’s clinical reference range for that target. The result of the manufacturer’s decision tree for *Lactobacillus* and the other reproductive-tract targets (optimal or non-optimal; Manual EndomeTRIO Custom OA, version 3.1, 2025) was analyzed separately (**Supplementary Table S6**). It was observed post hoc that among the CE-associated pathogen targets the two definitions identified the same women, because every value reported already exceeded its reference limit, whereas among the other reproductive-tract targets they did not (37 women detected versus 24 above the reference range). Because qPCR detects microbe-derived DNA rather than viable organisms, a positive result indicates target DNA and does not by itself confirm active infection or bacterial viability. The manufacturer’s report classifies the panel targets into three groups: *Lactobacillus*, bacteria most commonly associated with chronic endometritis, and other potentially pathogenic bacteria not related to chronic endometritis. Our three prespecified categories correspond to these three groups. The term non-*Lactobacillus*-dominant microbiota is not used by the manufacturer and is not applied to any category in this study. The 10 targets in the first group correspond closely to the organisms listed as classically implicated in CE by McQueen et al. (1, 6) and recovered from CE cohorts by Cicinelli et al. (9, 16) and Moreno et al. (25). The group assignment, basis for inclusion, unit of report and manufacturer reference range for every target are given in **Supplementary Table S3**.

All panel targets are reported in the same unit (genome copies per mL), but amplification efficiencies are not published, so copy numbers from different primer-probe sets are not on a validated common scale; the panel also includes no universal bacterial quantification, so no denominator representing the whole community is available. Relative abundance and *Lactobacillus* proportion therefore could not be assessed, and the second category was analyzed as detection of individual bacterial targets rather than as a measure of non-*Lactobacillus*-dominant microbiota. The manufacturer states that the assay reports absolute quantities rather than percentages, because the same percentage may correspond to different absolute quantities in samples of differing biomass; relative composition is therefore not part of the reported output by design. A summed bacterial load was not constructed, because all target-positive women had exactly one CE-associated target detected and copy numbers from different assays are not on a validated common scale. All three categories were analyzed as binary detection variables, and the manufacturer’s reference-range classification was analyzed separately.

### Hysteroscopy and incidental polyps

All women in the analysis underwent diagnostic hysteroscopy. Because this cohort was not selected for ultrasound-suspected polyps, hysteroscopy functioned as incidental screening. Identified polyps were classified as micro, single, or multiple. To assess whether incidental polyps confound the microbiota–CD138 association, the primary analysis was repeated in two prespecified sensitivity analyses: (a) exclusion of single + multiple polyps (exclusion of pure, microbiota-independent competing causes) and (b) exclusion of all polyps including micropolyps (because micropolyps are a finding accompanying CE, their exclusion is not strictly necessary, but this was performed as a more conservative sensitivity analysis).

Because endometriosis and adenomyosis may generate a local inflammatory environment independently of the endometrial microbiota (23, 24), two post hoc sensitivity analyses were performed alongside the polyp-exclusion analyses: (c) exclusion of all women with endometriosis or adenomyosis, and (d) exclusion of these women together with all polyps.

### Statistical analysis

Each qPCR category was dichotomized (detected/not detected), and its association with CD138 positivity was tested using Fisher’s exact test. Unadjusted odds ratios (ORs) were calculated as the cross-product ratio, applying a Haldane–Anscombe 0.5 correction when any cell was 0, with 95% confidence intervals (CIs) estimated by the logit (Woolf) method. Sensitivity, specificity, positive predictive value (PPV), and negative predictive value (NPV) were calculated using CD138 as the reference. Detection rates were also examined by CD138 score. Associations between individual bacterial targets and CD138 positivity were also assessed using Fisher’s exact test. Target-level analyses were exploratory and were corrected post hoc by the Benjamini-Hochberg false discovery rate and by the Holm procedure within each prespecified panel family, that is across the 10 CE-associated targets and across the 16 other reproductive-tract targets, including targets that were never detected; results for the broader family of all 26 non-*Lactobacillus* targets are also reported. *Lactobacillus* was one of the three prespecified category-level exposures and was therefore not included in the target-level correction. The three prespecified category-level comparisons were not corrected for multiplicity. Because only 13 women were target-positive, the primary association was additionally estimated by Firth penalized logistic regression and by the exact conditional odds ratio. Continuous variables were compared using Mann–Whitney U test. A two-sided P value of <0.05 was considered statistically significant. Odds ratios are cross-product ratios with logit (Woolf) confidence intervals whereas P values are from the exact test; because these are computed by different methods, a confidence interval that marginally excludes 1 may accompany an exact P value above 0.05 in small strata, and inference was based on the exact test throughout. Analyses were performed in Python (pandas, SciPy). In addition, CD138 positivity rates were compared among the three sampling-timing groups using chi-square test, and the main pathogen–CD138 association was evaluated using logistic regression adjusted for sampling timing.

A multivariable logistic regression model was also fitted, with CD138 positivity as the dependent variable and pathogen-target detection, other reproductive-tract bacterial target detection, *Lactobacillus* detection, any incidental polyp, endometriosis, adenomyosis, and sampling timing as independent variables, together with a parsimonious model containing pathogen-target detection, any incidental polyp, and a combined endometriosis or adenomyosis term. The multivariable analysis and the endometriosis or adenomyosis analyses were post hoc and were not corrected for multiplicity.

## Results

### Cohort characteristics

From the source population, no women were excluded because of antibiotic use within 1 month before sampling; 6 probiotic users, 10 women in whom CD138 could not be assessed, and 3 women without hysteroscopy were excluded, leaving 126 women for analysis (**Figure 1**). Their mean age was 37.1 years; 22 women had RPL (≥2 miscarriages) and 43 had RIF (≥2 failed transfers) (**Supplementary Table S1**). CD138-positive endometrium (CE) was found in 57 of 126 women (45.2%). Among CD138-positive women, the most common plasma cell score was 1, with score 2 observed in 9 women and score 3 in 3 women, indicating that much of the histologic CE in this infertile population was of low plasma cell density. Background factors such as age, BMI, RPL/RIF history, adenomyosis, and endometriosis did not differ significantly between the CD138-positive and CD138-negative groups (**Table 1**). The prevalence of adenomyosis (1.8% vs. 5.8%, P = 0.377) and endometriosis (12.3% vs. 7.2%, P = 0.375) also did not differ significantly between the groups. Incidental polyps were identified via hysteroscopy in 20 women (15.9%) and were significantly more frequent in the CD138-positive group than in the CD138-negative group (14/57 [24.6%] vs. 6/69 [8.7%], P = 0.026; **Table 1**). By type, there were 13 micro, 4 single, and 4 multiple polyps. Because one woman had coexisting micro and multiple polyps, the type-specific total was 21 against 20 women. Since polyps are a known, microbiota-independent cause of CD138 positivity (micropolyps being regarded as a finding accompanying CE) and showed an uneven distribution, their confounding was assessed in two prespecified sensitivity analyses.

All endometrial samples were obtained in the secretory phase; no proliferative-phase samples were included. CD138 positivity rates were 43.5% (40/92), 40.0% (6/15), and 57.9% (11/19) in the early secretory, mid-secretory, and late secretory phases, respectively, with no significant difference among the three groups (P = 0.47). The distribution of sampling timing also did not differ between the CD138-positive and CD138-negative groups (P = 0.47; **Table 1**). The association between CE-associated pathogen-target detection and CD138 positivity was maintained after adjustment for sampling timing (crude OR 8.01 → timing-adjusted OR 7.53, 95% CI 1.58–35.84, P = 0.011; **Supplementary Table S2**).

### Association of qPCR categories with CD138 positivity

CE-associated pathogen targets were detected in 13 women (10.3%), other reproductive-tract bacterial targets in 37 (29.4%), and *Lactobacillus* in 70 (55.6%). Of the three prespecified categories, only detection of CE-associated pathogen targets was significantly associated with CD138 positivity (OR 8.01, 95% CI 1.70–37.85, P = 0.003). Neither other reproductive-tract bacterial targets (OR 0.55, 95% CI 0.25–1.22, P = 0.171) nor *Lactobacillus* detection (OR 0.54, 95% CI 0.27–1.11, P = 0.107) was significantly associated with CD138 positivity (**Figure 2**). Detection of CE-associated pathogen targets showed high specificity (97.1%) and PPV (84.6%) for CD138-positive CE, but sensitivity was low at 19.3%. Of 13 pathogen-target-positive women, 11 (84.6%) were CD138-positive, whereas 46 of 57 CD138-positive women (80.7%) had no detectable pathogen (**Supplementary Figure S1**). Classification metrics for each category against CD138 positivity are shown in **Figure 2**. Because only 13 women were target-positive, the primary association was re-estimated with methods robust to sparse data: the exact conditional odds ratio was 7.89 (95% CI 1.61-76.56) and the Firth penalized odds ratio was 6.68 (95% CI 1.54-28.99), rising to 7.40 (95% CI 1.67-32.90) after adjustment for any incidental polyp and for endometriosis or adenomyosis. The point estimate was therefore stable across estimators, whereas the exact interval makes the imprecision explicit.

**Figure 2:**
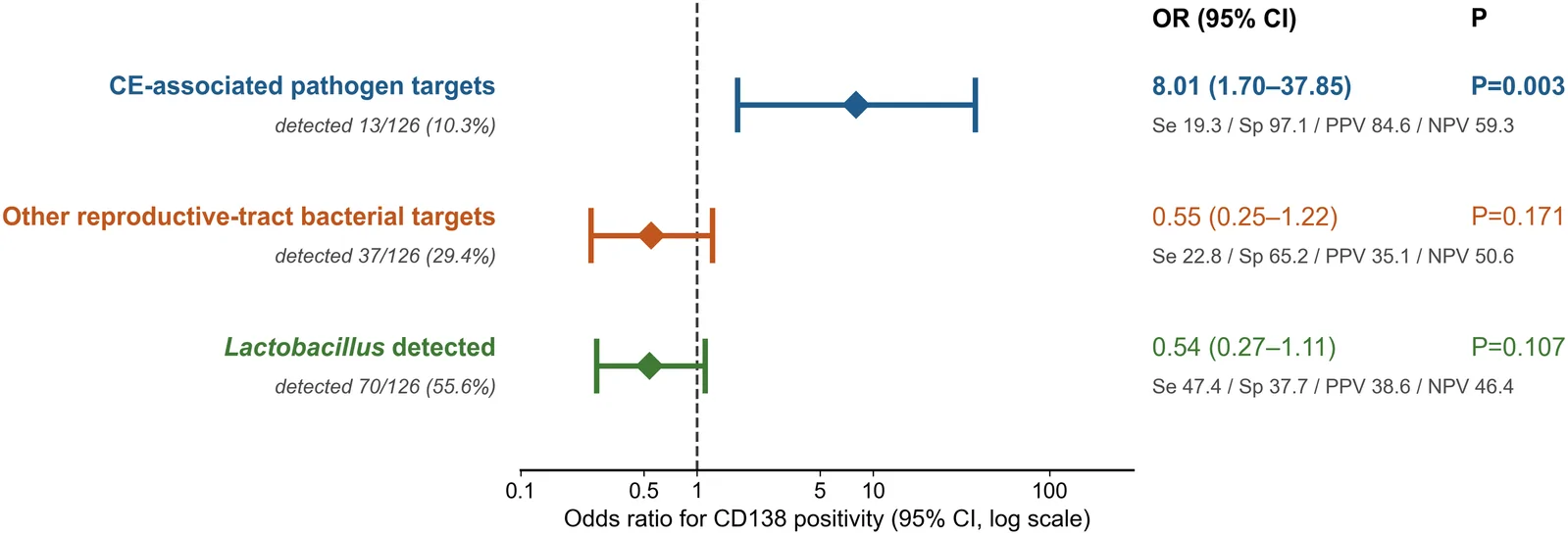
Association between CD138 positivity and each qPCR category (N = 126). Only detection of CE-associated pathogen targets is significantly and positively associated. The dashed line indicates OR = 1. Detection denotes qPCR amplification of target DNA at a Crt of 28 or less (the platform detection cut-off) and does not establish bacterial viability or active infection. The second category represents detection of individual bacterial targets that the manufacturer classifies as potentially pathogenic bacteria not related to chronic endometritis; it is not a measure of non-*Lactobacillus*-dominant microbiota. Se, sensitivity; Sp, specificity; PPV, positive predictive value; NPV, negative predictive value.

### Plasma cell burden and species-level findings

As the CD138 score increased, the detection rate of CE-associated pathogen targets increased from 2.9% at score 0 to 17.8%, 11.1%, and 66.7% at scores 1, 2, and 3, respectively, whereas other reproductive-tract bacterial targets (34.8% → 33.3%) and *Lactobacillus* detection (62.3% → 0%) did not increase with plasma cell burden (**Supplementary Figure S2**). However, because score 3 was detected in only 3 women, this is a suggestive finding only. At the target level, *Escherichia coli* was detected in 5 women, all of whom were CD138-positive (uncorrected P = 0.017); after correction for multiple comparisons within the prespecified family of 10 CE-associated targets this association was no longer significant (Benjamini-Hochberg and Holm P = 0.171; P = 0.446 in the broader family of all 26 non-*Lactobacillus* targets), and it should be regarded as hypothesis-generating only. Individual other reproductive-tract bacterial targets (most frequently *Bifidobacterium* spp., *Gardnerella vaginalis*, and *Atopobium vaginae*) were not associated with CD138 positivity (**Supplementary Table S3**). Binary detection of the genus *Lactobacillus* was not associated with CD138 positivity (odds ratio 0.54, 95% CI 0.27-1.11, P = 0.107), and no *Lactobacillus* species was significantly associated with CD138 positivity, although the point estimates differed in direction between species (*L. crispatus* 0.43, *L. iners* 1.01, *L. jensenii* 1.91; **Supplementary Table S5**, **Supplementary Figure S3**). An unadjusted post hoc comparison of reported *Lactobacillus* values including women with no detectable *Lactobacillus* gave P = 0.045; the higher proportion of women with no detectable *Lactobacillus* in the CD138-positive group contributed to this difference, and the comparison restricted to the 70 *Lactobacillus*-positive women was not significant (P = 0.242). Taken together, these analyses are directionally consistent but not confirmatory. CD138 positivity was 53.6%, 48.6% and 28.6% across the three strata of reported *Lactobacillus* values (undetected, below median, and at or above median; chi-square P = 0.059), and the adjusted odds ratio per 10-fold increase in the reported *Lactobacillus* value was 0.84 (95% CI 0.67-1.05, P = 0.13); the cut-off was data-derived and these post hoc analyses are exploratory. By the manufacturer’s decision tree for *Lactobacillus* and the other reproductive-tract targets, CD138 positivity was 41.3% in women with an optimal result, 29.2% in women classified as non-optimal because at least one other reproductive-tract target was out of its reference range, and 61.5% in women classified as non-optimal because *Lactobacillus* was not detected (chi-square P = 0.029; **Supplementary Table S6**).

### Robustness to incidental polyps

The association between CE-associated pathogen targets and CD138 positivity was maintained after exclusion of incidental polyps and was numerically stronger, although the confidence intervals were wide because of the small number of pathogen-target-positive cases (after exclusion of single + multiple polyps, OR 17.44 [95% CI 2.17–140.17, P < 0.001]; after exclusion of all polyps including micropolyps, OR 18.79 [95% CI 2.30–153.21, P < 0.001]). In both sensitivity analyses, other reproductive-tract bacterial targets and *Lactobacillus* detection showed no significant association with CD138 positivity (**Figure 3**). This indicates that incidental polyps did not explain the pathogen–CE association but rather diluted it in the whole cohort.

**Figure 3:**
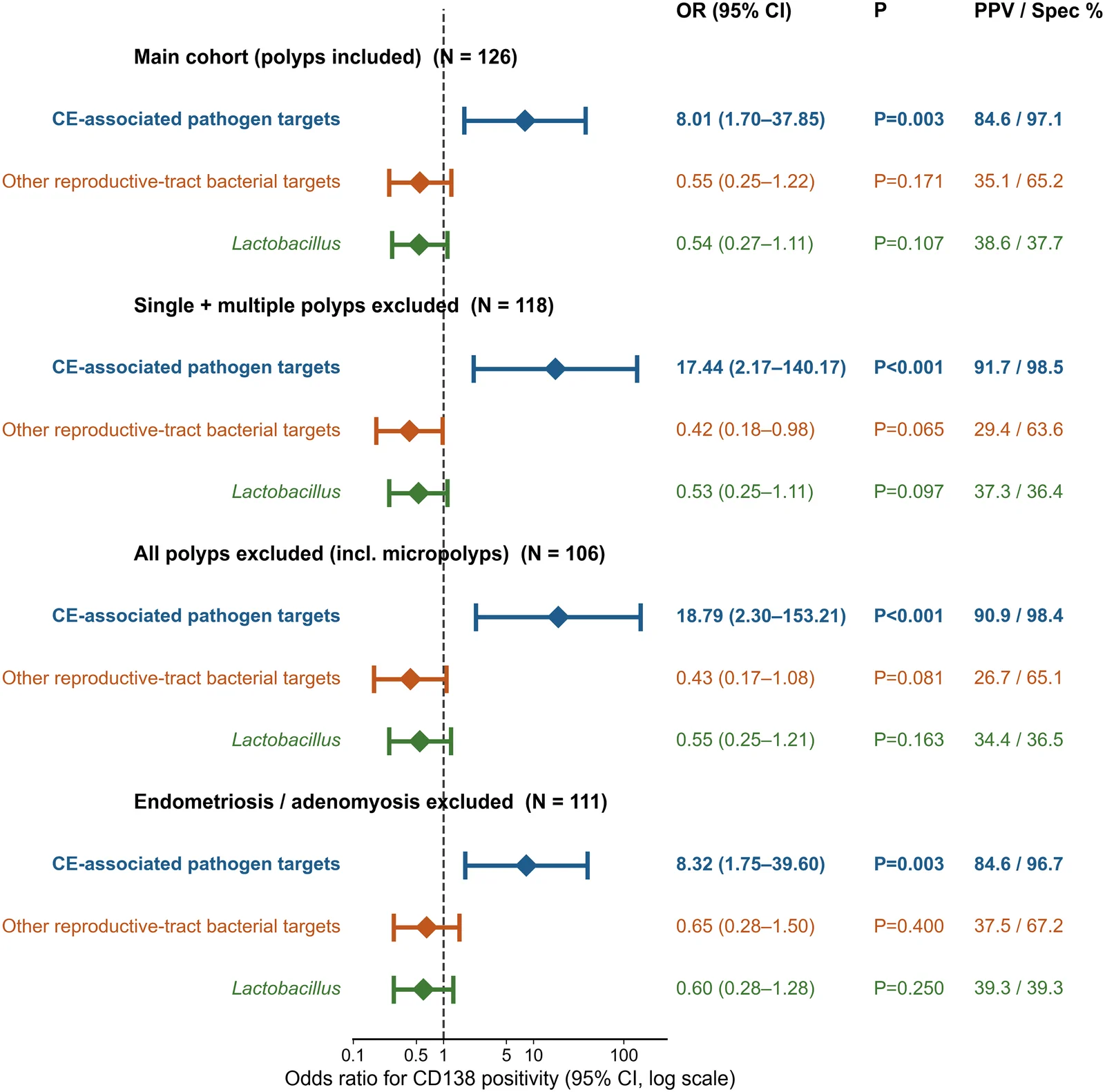
Sensitivity analyses. Odds ratios for CD138 positivity are shown for each qPCR category in four row groups: the main cohort (n = 126); single and multiple polyps excluded (n = 118), which removes purely anatomical competing causes; all polyps including micropolyps excluded (n = 106), a stricter analysis that also removes micropolyps, which are regarded as a phenotype of CE; and women with endometriosis or adenomyosis excluded (n = 111), which addresses local inflammation of non-microbial origin. The association between CE-associated pathogen targets and CD138 positivity was maintained in every group, whereas other reproductive-tract bacterial targets and *Lactobacillus* remained nonsignificant throughout. The dashed line indicates an odds ratio of 1, and the horizontal bars are 95% confidence intervals on a logarithmic scale. The two additional sensitivity analyses, in which women with endometriosis or adenomyosis were excluded together with single and multiple polyps (n = 103) or with all polyps including micropolyps (n = 94), are reported in **Supplementary Table S4**. Detection denotes qPCR amplification of target DNA at a Crt of 28 or less (the platform detection cut-off) and does not establish bacterial viability or active infection. The second category represents detection of individual bacterial targets that the manufacturer classifies as potentially pathogenic bacteria not related to chronic endometritis; it is not a measure of non-*Lactobacillus*-dominant microbiota. PPV, positive predictive value; Spec, specificity.

### Robustness to endometriosis and adenomyosis, and multivariable analysis

Endometriosis was recorded in 12 women (9.5%) and adenomyosis in 5 (4.0%); 15 women (11.9%) had either condition (2 women had both), and CD138 positivity did not differ between women with and without these conditions (7/15 [46.7%] vs. 50/111 [45.0%]). After exclusion of all affected women (n = 111), the association between CE-associated pathogen detection and CD138 positivity was essentially unchanged (OR 8.32, 95% CI 1.75-39.60, P = 0.003), and neither other reproductive-tract bacterial targets (OR 0.65, 95% CI 0.28-1.50, P = 0.400) nor *Lactobacillus* (OR 0.60, 95% CI 0.28-1.28, P = 0.250) was associated with CD138 positivity (**Figure 3**). When these women and all polyps were excluded simultaneously (n = 94), the pathogen association was again numerically stronger (OR 18.62, 95% CI 2.27-152.76, P < 0.001). All sensitivity analyses are summarized in **Supplementary Table S4**.

In the full multivariable model, detection of CE-associated pathogen targets remained independently associated with CD138 positivity (adjusted OR 7.89, 95% CI 1.55-40.06, P = 0.013), as did any incidental polyp (adjusted OR 3.80, 95% CI 1.26-11.49, P = 0.018), whereas other reproductive-tract bacterial targets (adjusted OR 0.42, 95% CI 0.17-1.07, P = 0.069), *Lactobacillus* (adjusted OR 0.62, 95% CI 0.27-1.40, P = 0.246), endometriosis (adjusted OR 2.49, 95% CI 0.62-10.03, P = 0.200), and adenomyosis (adjusted OR 0.48, 95% CI 0.04-5.47, P = 0.551) were not. In the parsimonious model, the adjusted OR for pathogen-target detection was 8.94 (95% CI 1.85-43.24, P = 0.006). Incidental polyps therefore behaved as an independent contributor to CD138 positivity rather than as a confounder of the pathogen association.

## Discussion

In this extended cohort with concurrent histology and qPCR and with hysteroscopic assessment, only detection of CE-associated pathogen targets was associated with CD138-defined CE, independent of incidental endometrial polyps. Other reproductive-tract bacterial targets were not associated with CD138 positivity in the primary or polyp-exclusion analyses, and *Lactobacillus* was not significantly associated, although the direction of the estimates was consistent across analyses. These findings separate a pathogen axis linked to histologic inflammation from the detection of individual other reproductive-tract bacterial targets, which was not associated with CD138 positivity.

The pathogen panel targets bacteria historically implicated in CE (1, 6, 9). All *Escherichia coli* detections occurred in CD138-positive endometrium, but this target-level association did not survive correction for multiple comparisons and is hypothesis-generating only. Throughout, detection denotes qPCR amplification of target DNA at a Crt of 28 or less (the platform detection cut-off); qPCR does not distinguish DNA from viable and non-viable organisms, so a positive result identifies a DNA-defined rather than a demonstrably infected subset. The high specificity (97.1%) and PPV (84.6%) of pathogen-target detection indicate that a positive qPCR result strongly predicts histologic CE, whereas the low sensitivity (19.3%) means that a negative result does not exclude CE.

The large pathogen-negative fraction of CD138-positive women (80.7%) suggests that much CD138-defined CE is not attributable to bacteria on the current panel. Possible explanations include immune-mediated or noninfectious inflammation, off-panel bacteria, and structural or inflammatory endometrial factors. CE has been reported more frequently in women with endometriosis and adenomyosis (23, 24), and sterile inflammation could produce CD138 positivity without detectable pathogens. This interpretation is consistent with the lack of association between CD138 positivity and either *Lactobacillus* or other reproductive-tract bacterial targets, with previous disagreement among histology, culture, hysteroscopy, and molecular methods (25), and with recent reports suggesting that dysbiosis testing and CE testing may capture distinct entities (26, 27). This may explain why next-generation sequencing studies, which report only relative abundance and thereby couple NLDM and *Lactobacillus* depletion, link microbiota composition to reproductive outcomes (12, 13), whereas absolute qPCR-based detection localizes the histologic signal to the pathogen axis.

Because pathogen-negative cases account for most CD138-positive endometrium, plasma-cell infiltration in this cohort cannot be read as a microbiological finding alone. Current syntheses place CD138-positive plasma cells alongside CD56-positive uterine natural killer cells and the local cytokine milieu as interacting elements of a single inflammatory phenotype (11), and distinguishing infection-driven from immune-driven CD138 positivity will require immune phenotyping of the endometrium rather than more sensitive microbiology. This also bears on the model in which a *Lactobacillus*-depleted community sustains a pro-inflammatory endometrial microenvironment (10): that model could not be tested here, because the panel provides no universal bacterial quantification and therefore no measure of community composition; what these data show is only that detection of individual other reproductive-tract targets was not associated with CD138-defined histology, which is consistent with dysbiosis and CD138-defined CE not being interchangeable readouts of a single process, but does not establish it. Similarly, endometrial polyps and CE have been proposed to represent consecutive phases of a common process (11), whereas our multivariable analysis, in which incidental polyps were associated with CD138 positivity independently of pathogen-target detection, is more consistent with two partly separate routes.

A methodological question raised by these findings is whether the absence of an association between other reproductive-tract bacterial targets or *Lactobacillus* and CD138 positivity reflects the use of binary detection. Eubiosis is conventionally defined by relative composition rather than by the presence or absence of any single organism, and we accept that binary detection is a coarser instrument for testing the dysbiosis hypothesis. Reports linking NLDM to CE do not usually separate three mechanistically distinct elements: the absence of detectable *Lactobacillus*, the relative balance between *Lactobacillus* and other organisms, and the presence of particular non-*Lactobacillus* organisms. The present design addresses the first and third directly, and neither was associated with CD138 positivity in any sensitivity analysis. The second element, however, is not one that this platform allows us to evaluate: because each target is amplified by a different primer-probe set and no universal bacterial quantification is included, copy numbers from different assays cannot be summed into a common denominator, and relative abundance cannot be derived from the reported values. We also note that the same binary framework detected a strong and reproducible association for the pathogen category in every sensitivity analysis, so the method is evidently able to resolve a microbiological signal when one is present. A study using universal 16S ribosomal RNA quantification, in which the denominator is the total bacterial load, would nevertheless be required to exclude a moderate association with relative *Lactobacillus* depletion. The closest community-level readout available to us is the manufacturer’s own decision tree for *Lactobacillus* and the other reproductive-tract targets, and it points the same way: CD138 positivity was not higher in women classified as non-optimal because another reproductive-tract target was out of its reference range (29.2%) than in women with an optimal result (41.3%), and was highest in women classified as non-optimal because *Lactobacillus* was not detected (61.5%; **Supplementary Table S6**). This is consistent with the interpretation offered by Moreno et al., who concluded that what may interfere with reproductive outcome is the presence of pathogens in the uterine cavity rather than the requirement for a specific commensal taxon, and who proposed that the principal role of *Lactobacillus* may be to prevent colonization of the cavity by pathogenic bacteria (13).

Clinically, endometrial microbiota composition testing should not be used as a surrogate for CD138-defined CE, and *Lactobacillus* depletion or an NLDM profile alone should not justify CE-targeted antimicrobial therapy. qPCR pathogen-target detection is best positioned as a high-specificity complement to, not a replacement for, CD138 histology and hysteroscopy. These findings also caution against interpreting CD138 positivity, particularly low-grade score 1 positivity, as evidence of a possible active bacterial component. The scoring threshold used here (McQueen score ≥1 per HPF) represents a higher plasma-cell density than the commonly used ≥1 per 10 HPFs, although hotspot-based assessment may increase detection compared with randomly selected fields. This difference should be considered when comparing the observed 45.2% prevalence with other studies. Consistent with our results, Klimaszyk et al. found no correlation between qPCR-based endometrial bacterial detection and CD138-defined CE in a recurrent pregnancy loss cohort (28).

These results also bear on the order in which the two tests should be used. Histologic and molecular evaluation are complementary rather than competing, and our multivariable analysis indicates that incidental polyps act as an independent, microbiota-unrelated route to CD138 positivity. We therefore suggest that anatomical lesions capable of producing plasma-cell infiltration, in particular endometrial polyps, should first be excluded at hysteroscopy and treated where present, and that CD138 immunostaining and endometrial qPCR should then be performed on the same cycle rather than sequentially, for three reasons. First, any interval between the two tests introduces a time lag during which the microbiological and the histologic state may diverge. Second, because qPCR does not distinguish viable from non-viable organisms, a molecular result obtained after treatment or after an unrecorded interval is difficult to interpret in isolation. Third, only concurrent evaluation makes it possible to identify the pathogen-negative, CD138-positive group in which inflammation is presumably induced by a route other than the organisms on the panel. Within such an algorithm, a positive pathogen result carries a high posterior probability of histologic CE (positive predictive value 84.6%, rising to 91.7% once anatomical polyps are excluded), which may inform, but does not by itself justify, a decision to treat, because detection of target DNA does not establish the presence of viable organisms or of active infection; a negative result has little exclusionary value; conversely, an isolated NLDM profile or *Lactobacillus*-depleted result should not by itself trigger CE-directed antimicrobial therapy.

The strengths of this study are the matched histology and qPCR data, use of targeted absolute detection unaffected by relative-abundance constraints, exclusion of recent antibiotic and probiotic users, universal hysteroscopy, and prespecified polyp-exclusion analyses. The limitations are the retrospective single-center design, small number of pathogen-target-positive events with wide CIs, possible contribution of off-panel bacteria, incomplete ascertainment of prior antibiotic/probiotic exposure, possible residual confounding from unmeasured uterine or inflammatory conditions, lack of systematic architectural scoring on H&E, and absence of reproductive outcome and treatment-response data. All samples were obtained in the secretory phase; however, CD138 positivity did not differ significantly by timing and the main association remained after timing adjustment. Additionally, POD 1 (oocyte retrieval) and P + 5 sampling represent biologically distinct endometrial environments, and residual confounding by sampling timing cannot be fully excluded. Prospective multicenter studies should link these qPCR categories to reproductive outcome and antimicrobial response, ideally in a randomized trial of treatment allocated on the basis of molecular and histologic findings.

With 126 women evaluated by concurrent histology and qPCR and by hysteroscopy, this cohort is larger than most published series comparing molecular and histologic diagnosis of CE, although the number of pathogen-target-positive women remained small and the confidence intervals correspondingly wide. Organisms outside the panel are not detected, and off-panel bacteria may therefore contribute to the pathogen-negative fraction of CD138-positive women; panel-level analytical performance and target-specific limits of detection are reported by the manufacturer (see Methods), but limits of quantification, amplification efficiencies, primer and probe sequences and the statistical derivation of the reference ranges are not disclosed. The external assurance for the platform is ISO and CLIA accreditation. CD138 can also be expressed on the basolateral membrane of endometrial epithelial cells, a recognized source of false positivity (11); only stromal plasma cells were scored, but interobserver agreement was not formally quantified and alternative markers such as MUM1 were not used. The endometriosis and adenomyosis analyses and the multivariable model were post hoc, were based on small numbers of affected women, and were not corrected for multiplicity; they should be read as supportive rather than confirmatory. Several panel targets, including *Streptococcus agalactiae*/viridans, *Enterococcus faecalis*, *Ureaplasma urealyticum*, *Mycoplasma hominis*, *Gardnerella vaginalis* and *Atopobium vaginae*, are common lower-genital-tract commensals, and a supra-threshold endometrial signal for these targets may reflect colonization or transit contamination rather than endometrial infection. Because the panel quantifies selected targets with separate assays and includes no universal bacterial quantification, its semiquantitative output cannot be treated as equivalent to the relative-abundance measures used in next-generation-sequencing-based assessment of dysbiosis; bacterial burden and community composition were therefore not analyzed. Endometrial culture was not performed as part of routine care in this cohort and no concurrent endometrial culture was available, so a comparison between qPCR and culture at the level of the endometrial cavity was not possible. Endometrial culture is not itself a reference standard: in the comparative study of Moreno et al. the concordance between molecular microbiology and endometrial culture was 56.92%, and histology, hysteroscopy and culture agreed in only 20% of women (25). In a low-biomass compartment a culture-negative result cannot demonstrate the absence of viable organisms, and a culture-positive result cannot distinguish active infection from colonization, transient passage or contamination at sampling. Future studies could assess whether the DNA-defined, pathogen-target-positive subset described here is accompanied by viable or metabolically active organisms and by endometrial inflammation, but no single test, and no degree of agreement between tests, can by itself establish a microbiological cause.

## Conclusion

In this retrospective cohort with concurrent CD138 immunostaining, endometrial qPCR, and hysteroscopy, detection of prespecified CE-associated pathogen targets was strongly associated with CD138-positive endometrium, whereas detection of individual bacterial targets commonly found in NLDM profiles and of *Lactobacillus* was not. Because the panel provides no total bacterial load and no universal denominator, these data cannot evaluate non-*Lactobacillus*-dominant microbiota as a community state, and no conclusion about it should be drawn from them. Because most CD138-positive cases were pathogen-negative, CD138-positive CE appears etiologically heterogeneous. qPCR pathogen-target detection may serve as a high-specificity complementary marker for a DNA-defined pathogen-associated subset of CD138-positive CE, but prospective studies linking qPCR findings to antimicrobial response and reproductive outcomes are needed.

## Data Availability

The original contributions presented in the study are included in the article/**Supplementary Material**. Further inquiries can be directed to the corresponding author.

## References

[1] McQueen DB Bernardi LA Stephenson MD . Chronic endometritis in women with recurrent early pregnancy loss and/or fetal demise. Fertil Steril. (2014) 101:1026–30. doi: 10.1016/j.fertnstert.2013.12.031 24462055

[2] Kitaya K . Prevalence of chronic endometritis in recurrent miscarriages. Fertil Steril. (2011) 95:1156–8. doi: 10.1016/j.fertnstert.2010.09.061 21030015

[3] Kasius JC Fatemi HM Bourgain C Sie-Go DM Eijkemans RJ Fauser BC . The impact of chronic endometritis on reproductive outcome. Fertil Steril. (2011) 96:1451–6. doi: 10.1016/j.fertnstert.2011.09.039 22019126

[4] Johnston-MacAnanny EB Hartnett J Engmann LL Nulsen JC Sanders MM Benadiva CA . Chronic endometritis is a frequent finding in women with recurrent implantation failure after *in vitro* fertilization. Fertil Steril. (2010) 93:437–41. doi: 10.1016/j.fertnstert.2008.12.131 19217098

[5] Bouet PE El Hachem H Monceau E Gariépy G Kadoch IJ Sylvestre C . Chronic endometritis in women with recurrent pregnancy loss and recurrent implantation failure: prevalence and role of office hysteroscopy and immunohistochemistry in diagnosis. Fertil Steril. (2016) 105:106–10. doi: 10.1016/j.fertnstert.2015.09.025 26456229

[6] McQueen DB Perfetto CO Hazard FK Lathi RB . Pregnancy outcomes in women with chronic endometritis and recurrent pregnancy loss. Fertil Steril. (2015) 104:927–31. doi: 10.1016/j.fertnstert.2015.06.044 26207958

[7] Bayer-Garner IB Nickell JA Korourian S . Routine syndecan-1 immunohistochemistry aids in the diagnosis of chronic endometritis. Arch Pathol Lab Med. (2004) 128:1000–3. doi: 10.5858/2004-128-1000-RSIAIT 15335255

[8] Kasius JC Broekmans FJM Sie-Go DMDS Bourgain C Eijkemans MJC Fatemi HM . The reliability of the histological diagnosis of endometritis in asymptomatic IVF cases: a multicenter observer study. Hum Reprod. (2012) 27:153–8. doi: 10.1093/humrep/der341 22025228

[9] Cicinelli E Matteo M Tinelli R Lepera A Alfonso R Indraccolo U . Prevalence of chronic endometritis in repeated unexplained implantation failure and the IVF success rate after antibiotic therapy. Hum Reprod. (2015) 30:323–30. doi: 10.1093/humrep/deu292 25385744

[10] Aquino CI Nicosia A Ligori A Volpicelli AI Surico D . Microbiota status and endometrial cancer: a narrative review about possible correlations in affected versus healthy patients. Sci. (2024) 6:75. doi: 10.3390/sci604007530654563

[11] Aquino CI Ligori A Tinelli R Boldorini R Cosma S Surico D . Narrative review on chronic endometritis and its diagnosis in the reproductive process: focus on possible future diagnostic perspectives. Sci. (2026) 8:167. doi: 10.3390/sci807016730654563

[12] Moreno I Codoñer FM Vilella F Valbuena D Martinez-Blanch JF Jimenez-Almazán J . Evidence that the endometrial microbiota has an effect on implantation success or failure. Am J Obstet Gynecol. (2016) 215:684–703. doi: 10.1016/j.ajog.2016.09.075 27717732

[13] Moreno I Garcia-Grau I Perez-Villaroya D Gonzalez-Monfort M Bahçeci M Barrionuevo MJ . Endometrial microbiota composition is associated with reproductive outcome in infertile patients. Microbiome. (2022) 10:1. doi: 10.1186/s40168-021-01184-w 34980280 PMC8725275

[14] Benner M Ferwerda G Joosten I van der Molen RG . How uterine microbiota might be responsible for a receptive, fertile endometrium. Hum Reprod Update. (2018) 24:393–415. doi: 10.1093/humupd/dmy012 29668899

[15] Liu Y Ko EL Wong KW Chen X Cheung WC Law TS . Endometrial microbiota in infertile women with and without chronic endometritis as diagnosed using a quantitative and reference range-based method. Fertil Steril. (2019) 112:707–717.e1. doi: 10.1016/j.fertnstert.2019.05.015 31327470

[16] Cicinelli E Matteo M Tinelli R Pinto V Marinaccio M Indraccolo U . Chronic endometritis due to common bacteria is prevalent in women with recurrent miscarriage as confirmed by improved pregnancy outcome after antibiotic treatment. Reprod Sci. (2014) 21:640–7. doi: 10.1177/1933719113508817 24177713 PMC3984485

[17] Kitaya K Matsubayashi H Takaya Y Nishiyama R Yamaguchi K Takeuchi T . Live birth rate following oral antibiotic treatment for chronic endometritis in infertile women with repeated implantation failure. Am J Reprod Immunol. (2017) 78:e12719. doi: 10.1111/aji.12719 28608596

[18] Vitagliano A Saccardi C Noventa M Di Spiezio Sardo A Saccone G Cicinelli E . Effects of chronic endometritis therapy on *in vitro* fertilization outcome in women with repeated implantation failure: a systematic review and meta-analysis. Fertil Steril. (2018) 110:103–112.e1. doi: 10.1016/j.fertnstert.2018.03.017 29908776

[19] Cicinelli E McQueen DB Huepfel B Vitagliano A Matteo M Davò R . Should patients be screened for chronic endometritis before assisted reproductive technology? Fertil Steril. (2022) 118:639–52. doi: 10.1016/j.fertnstert.2022.08.846 36182261

[20] Vitagliano A Cialdella M Cicinelli R Santarsiero CM Greco P Buzzaccarini G . Association between endometrial polyps and chronic endometritis: is it time for a paradigm shift in the pathophysiology of endometrial polyps in pre-menopausal women? Results of a systematic review and meta-analysis. Diagnostics. (2021) 11:2182. doi: 10.3390/diagnostics11122182 34943419 PMC8700534

[21] Winters AD Romero R Gervasi MT Gomez-Lopez N Tran MR Garcia-Flores V . Does the endometrial cavity have a molecular microbial signature? Sci Rep. (2019) 9:9905. doi: 10.1038/s41598-019-46173-0 31289304 PMC6616349

[22] Salter SJ Cox MJ Turek EM Calus ST Cookson WO Moffatt MF . Reagent and laboratory contamination can critically impact sequence-based microbiome analyses. BMC Biol. (2014) 12:87. doi: 10.1186/s12915-014-0087-z 25387460 PMC4228153

[23] Cicinelli E Trojano G Mastromauro M Vimercati A Marinaccio M Mitola PC . Higher prevalence of chronic endometritis in women with endometriosis: a possible etiopathogenetic link. Fertil Steril. (2017) 108:289–295.e1. doi: 10.1016/j.fertnstert.2017.05.016 28624114

[24] Takebayashi A Kimura F Kishi Y Ishida M Takahashi A Yamanaka A . The association between endometriosis and chronic endometritis. PloS One. (2014) 9:e88354. doi: 10.1371/journal.pone.0088354 24558386 PMC3928198

[25] Moreno I Cicinelli E Garcia-Grau I Gonzalez-Monfort M Bau D Vilella F . The diagnosis of chronic endometritis in infertile asymptomatic women: a comparative study of histology, microbial cultures, hysteroscopy, and molecular microbiology. Am J Obstet Gynecol. (2018) 218:602.e1–602.e16. doi: 10.1016/j.ajog.2018.02.012 29477653

[26] Hiratsuka D Matsuo M Kashiwabara K Inoue M Ishizawa C Iida R . Comparison of diagnostic tests for chronic endometritis and endometrial dysbiosis in recurrent implantation failure: impact on pregnancy outcomes. Sci Rep. (2025) 15:8272. doi: 10.1038/s41598-025-92906-9 40065148 PMC11893751

[27] Hiratsuka D Matsuo M Hirota Y . The reproductive tract microbiome and female fertility: dysbiosis, disease links, and emerging therapeutic strategies. Fertil Steril. (2026) 125:574–82. doi: 10.1016/j.fertnstert.2026.01.010 41577072

[28] Klimaszyk K Wirstlein P Bednarek-Rajewska K Jankowski M Svarre Nielsen H Wender-Ożegowska E . Endometrial factors and pregnancy loss frequency in recurrent pregnancy loss patients: comparing RT-PCR microbiology, microbial cultures, and immunohistochemistry of endometrium biopsy. J Appl Genet. (2025) 66:459–68. doi: 10.1007/s13353-025-00949-5 39976842 PMC12000213

[29] Igenomix . Limit of Detection for Targets Present at EMMA & ALICE Tests (2025). Available online at: https://www.igenomix.com/lodea/ (Accessed September 12, 2026).

