# Supplementary material for: Detection of prespecified CE-associated pathogen targets by quantitative polymerase chain reaction is associated with CD138-positive endometrium: a retrospective cohort study
Source: Front Immunol. 2026 Oct 13;17:1928249. doi: 10.3389/fimmu.2026.1928249 (PMC13710246; doi:10.3389/fimmu.2026.1928249)
Supplement: Supplementary file 1 [file Image3.pdf]

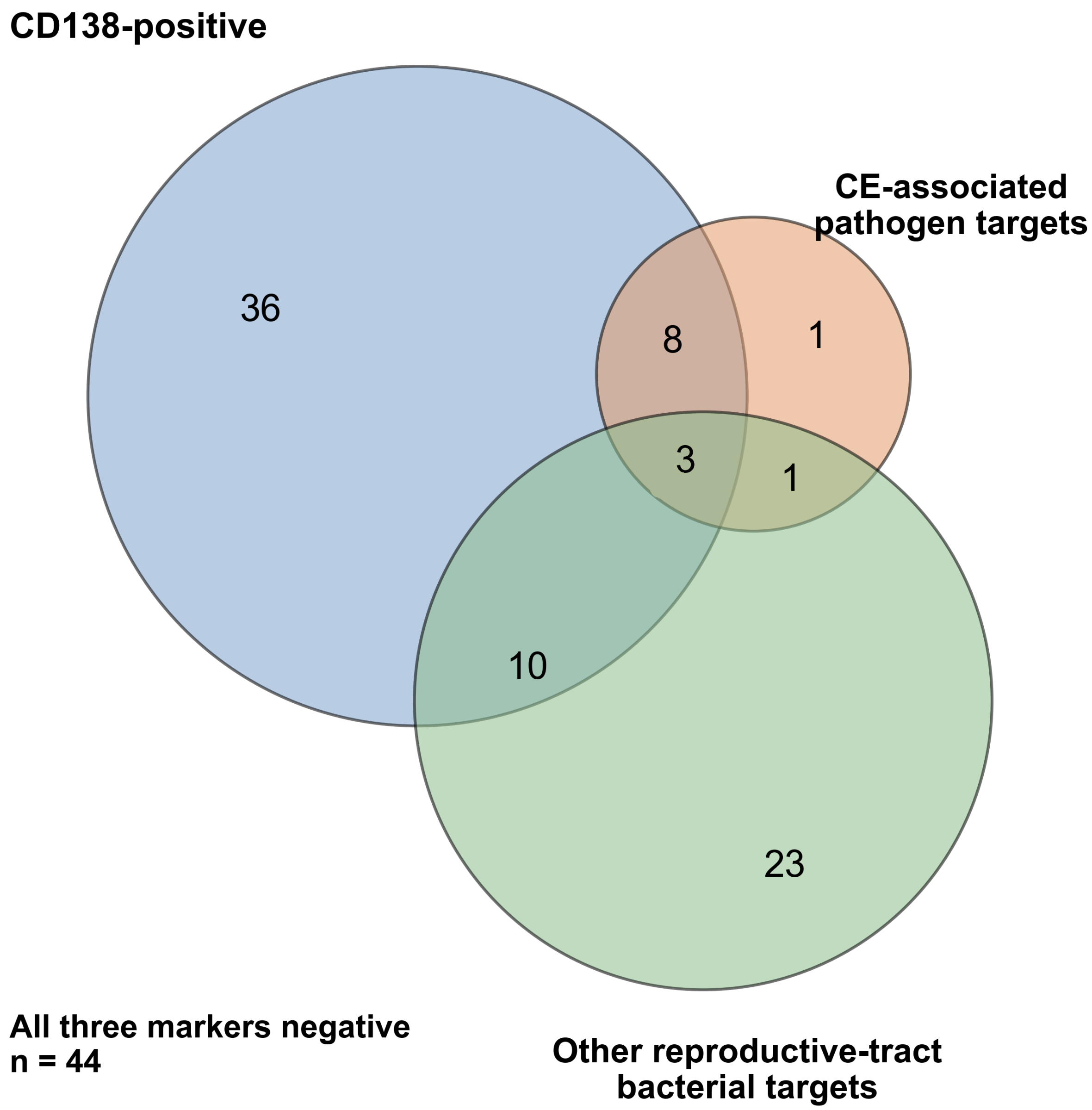
CD138-positive
CE-associated
pathogen targets
36
8
1
3
1
10
23
All three markers negative
n = 44
Other reproductive-tract
bacterial targets
