# Supplementary material for: Detection of prespecified CE-associated pathogen targets by quantitative polymerase chain reaction is associated with CD138-positive endometrium: a retrospective cohort study
Source: Front Immunol. 2026 Oct 13;17:1928249. doi: 10.3389/fimmu.2026.1928249 (PMC13710246; doi:10.3389/fimmu.2026.1928249)
Supplement: Supplementary file 2 [file DataSheet1.docx]

**Supplementary Material**

# Supplementary figure legends

**Supplementary Figure S1. Overlap between CD138 positivity, CE-associated pathogen targets and other reproductive-tract bacterial targets (N = 126).**

*Numbers are women in each region. Forty-four women were negative for all three markers. Detection denotes qPCR amplification of target DNA at a Crt of 28 or less (the platform detection cut-off) and does not establish bacterial viability or active infection. The second category represents detection of individual bacterial targets that the manufacturer classifies as potentially pathogenic bacteria not related to chronic endometritis; it is not a measure of non-Lactobacillus-dominant microbiota.*

**Supplementary Figure S2. Detection rate of each qPCR category by CD138 (plasma cell) score.**

*Score 3 was present in only 3 women, so the apparent rise at score 3 should be read as suggestive only. Detection denotes qPCR amplification of target DNA at a Crt of 28 or less (the platform detection cut-off) and does not establish bacterial viability or active infection. The second category represents detection of individual bacterial targets that the manufacturer classifies as potentially pathogenic bacteria not related to chronic endometritis; it is not a measure of non-Lactobacillus-dominant microbiota.*

**Supplementary Figure S3. Association between Lactobacillus detection and CD138 positivity, at genus level and for each species reported by the assay (N = 126).**

*Odds ratios with 95% confidence intervals on a logarithmic scale; the dashed line indicates an odds ratio of 1. No estimate reached statistical significance. The point estimates nevertheless differ in direction between species, with L. crispatus and L. gasseri below 1 and L. jensenii above 1, whereas the genus-level estimate averages these species into a single value. This illustrates why genus-level binary detection cannot test the hypothesis that Lactobacillus depletion is related to CD138-defined chronic endometritis.*

# Supplementary tables

**Supplementary Table S1. Exploratory subgroup analysis by clinical indication (RPL/RIF).**

| **Subgroup** | **n** | **CD138+ n (%)** | **Pathogen target+ n (%)** | **CD138+ among target+** |
| --- | --- | --- | --- | --- |
| RPL only (≥2 miscarriages, failed transfers <2) | 16 | 6 (37.5) | 1 (6.3) | 1/1 |
| RIF only (failed transfers ≥2, miscarriages <2) | 37 | 18 (48.6) | 2 (5.4) | 2/2 |
| RPL + RIF | 6 | 2 (33.3) | 1 (16.7) | 1/1 |
| Neither | 67 | 31 (46.3) | 9 (13.4) | 7/9 |

*Subgroups are exploratory and underpowered; no formal between-subgroup testing was performed.*

**Supplementary Table S2. CD138 positivity and CE-associated pathogen target detection by sampling timing.**

| **Sampling-timing subgroup** | **n** | **CD138+ n (%)** | **CE-associated pathogen target+ n (%)** |
| --- | --- | --- | --- |
| Early secretory (POD 1–5) | 92 | 40 (43.5) | 8 (8.7) |
| Mid-secretory (POD 6–8) | 15 | 6 (40.0) | 1 (6.7) |
| Late secretory (POD 9–13) | 19 | 11 (57.9) | 4 (21.1) |
| Total | 126 | 57 (45.2) | 13 (10.3) |

*Three-group comparison P = 0.47; the association was maintained after adjustment for sampling timing (OR 7.53, 95% CI 1.58–35.84, P = 0.011).*

**Supplementary Table S3. Complete panel-level results: basis for inclusion, manufacturer-reported limit of detection, quantitative endometrial qPCR output and association with CD138 positivity for all 27 targets (N = 126).**

| **Group** | **Target** | **Basis for inclusion (reference)** | **Reference range (GC/mL)** | LOD (genomic copies/µL) | **Detected, n (%)** | **CD138+ (n=57), n (%)** | **CD138- (n=69), n (%)** | **Median GC/mL** | **Range GC/mL** | **OR (95% CI)** | **P** | **P (BH)** | **P (Holm)** |
| --- | --- | --- | --- | --- | --- | --- | --- | --- | --- | --- | --- | --- | --- |
| CE-associated | Chlamydia trachomatis | Classically implicated in CE (1, 6); recovered from CE cohorts (9, 16, 25) | Absent (any detection) | 8 | 1 (0.8) | 1 (1.8) | 0 (0.0) | 1,230,000 | 1,230,000-1,230,000 | 3.69 (0.15-92.35) | 0.452 | 0.905 | 1.000 |
| CE-associated | Enterococcus faecalis | Classically implicated in CE (1, 6); recovered from CE cohorts (9, 16, 25) | 0-143 | 16 | 1 (0.8) | 1 (1.8) | 0 (0.0) | 360 | 360-360 | 3.69 (0.15-92.35) | 0.452 | 0.905 | 1.000 |
| CE-associated | Escherichia coli | Classically implicated in CE (1, 6); recovered from CE cohorts (9, 16, 25) | 0-230 | 8 | 5 (4.0) | 5 (8.8) | 0 (0.0) | 1,738 | 312-42,870 | 14.56 (0.79-269.24) | 0.017 | 0.171 | 0.171 |
| CE-associated | Klebsiella pneumoniae | Classically implicated in CE (1, 6); recovered from CE cohorts (9, 16, 25) | 0-247 | 80 | 0 (0.0) | 0 (0.0) | 0 (0.0) | - | - | - | - | - | - |
| CE-associated | Mycoplasma genitalium | Classically implicated in CE (1, 6); recovered from CE cohorts (9, 16, 25) | 0-123 | 160 | 0 (0.0) | 0 (0.0) | 0 (0.0) | - | - | - | - | - | - |
| CE-associated | Mycoplasma hominis | Classically implicated in CE (1, 6); recovered from CE cohorts (9, 16, 25) | 0-125 | 80 | 1 (0.8) | 0 (0.0) | 1 (1.4) | 10,532 | 10,532-10,532 | 0.40 (0.02-9.94) | 1.000 | 1.000 | 1.000 |
| CE-associated | Neisseria gonorrhoeae | Classically implicated in CE (1, 6); recovered from CE cohorts (9, 16, 25) | Absent (any detection) | 1.6 | 0 (0.0) | 0 (0.0) | 0 (0.0) | - | - | - | - | - | - |
| CE-associated | Staphylococcus aureus | Classically implicated in CE (1, 6); recovered from CE cohorts (9, 16, 25) | 0-219 | 16 | 1 (0.8) | 1 (1.8) | 0 (0.0) | 406 | 406-406 | 3.69 (0.15-92.35) | 0.452 | 0.905 | 1.000 |
| CE-associated | Streptococcus agalactiae (GBS)/S. viridans | Classically implicated in CE (1, 6); recovered from CE cohorts (9, 16, 25) | 0-91 | 400 / 80 | 4 (3.2) | 3 (5.3) | 1 (1.4) | 454 | 176-564 | 3.78 (0.38-37.35) | 0.328 | 0.905 | 1.000 |
| CE-associated | Ureaplasma urealyticum | Classically implicated in CE (1, 6); recovered from CE cohorts (9, 16, 25) | 0-292 | 16 | 0 (0.0) | 0 (0.0) | 0 (0.0) | - | - | - | - | - | - |
| Other reproductive-tract | Actinomyces israelii | Classified by the manufacturer as potentially pathogenic bacteria not related to CE; observed in endometrial profiles by 16S sequencing (12, 13) | Absent (any detection) | 1.6 | 0 (0.0) | 0 (0.0) | 0 (0.0) | - | - | - | - | - | - |
| Other reproductive-tract | Atopobium vaginae | Classified by the manufacturer as potentially pathogenic bacteria not related to CE; observed in endometrial profiles by 16S sequencing (12, 13) | 0-620 | 80 | 15 (11.9) | 5 (8.8) | 10 (14.5) | 7,222 | 786-165,000 | 0.57 (0.18-1.77) | 0.412 | 1.000 | 1.000 |
| Other reproductive-tract | Bacteroides fragilis | Classified by the manufacturer as potentially pathogenic bacteria not related to CE; observed in endometrial profiles by 16S sequencing (12, 13) | 0-526 | 80 | 0 (0.0) | 0 (0.0) | 0 (0.0) | - | - | - | - | - | - |
| Other reproductive-tract | Bifidobacterium spp. | Classified by the manufacturer as potentially pathogenic bacteria not related to CE; observed in endometrial profiles by 16S sequencing (12, 13) | 0-1,674 | 1.6 | 27 (21.4) | 10 (17.5) | 17 (24.6) | 1,438 | 170-355,000 | 0.65 (0.27-1.56) | 0.387 | 1.000 | 1.000 |
| Other reproductive-tract | Clostridium sordellii | Classified by the manufacturer as potentially pathogenic bacteria not related to CE; observed in endometrial profiles by 16S sequencing (12, 13) | Absent (any detection) | 1.6 | 0 (0.0) | 0 (0.0) | 0 (0.0) | - | - | - | - | - | - |
| Other reproductive-tract | Fusobacterium nucleatum | Classified by the manufacturer as potentially pathogenic bacteria not related to CE; observed in endometrial profiles by 16S sequencing (12, 13) | Absent (any detection) | 80 | 0 (0.0) | 0 (0.0) | 0 (0.0) | - | - | - | - | - | - |
| Other reproductive-tract | Gardnerella vaginalis | Classified by the manufacturer as potentially pathogenic bacteria not related to CE; observed in endometrial profiles by 16S sequencing (12, 13) | 0-982 | 0.3 | 16 (12.7) | 5 (8.8) | 11 (15.9) | 4,405 | 480-425,000 | 0.51 (0.17-1.56) | 0.288 | 1.000 | 1.000 |
| Other reproductive-tract | Haemophilus ducreyi | Classified by the manufacturer as potentially pathogenic bacteria not related to CE; observed in endometrial profiles by 16S sequencing (12, 13) | Absent (any detection) | 80 | 0 (0.0) | 0 (0.0) | 0 (0.0) | - | - | - | - | - | - |
| Other reproductive-tract | Mycobacterium tuberculosis | Classified by the manufacturer as potentially pathogenic bacteria not related to CE; observed in endometrial profiles by 16S sequencing (12, 13) | Absent (any detection) | 80 | 0 (0.0) | 0 (0.0) | 0 (0.0) | - | - | - | - | - | - |
| Other reproductive-tract | Mobiluncus spp. | Classified by the manufacturer as potentially pathogenic bacteria not related to CE; observed in endometrial profiles by 16S sequencing (12, 13) | 0-134 | 16 | 2 (1.6) | 0 (0.0) | 2 (2.9) | 454 | 432-476 | 0.23 (0.01-4.99) | 0.501 | 1.000 | 1.000 |
| Other reproductive-tract | Peptostreptococcus anaerobius | Classified by the manufacturer as potentially pathogenic bacteria not related to CE; observed in endometrial profiles by 16S sequencing (12, 13) | 0-171 | 80 | 0 (0.0) | 0 (0.0) | 0 (0.0) | - | - | - | - | - | - |
| Other reproductive-tract | Porphyromonas asaccharolytica | Classified by the manufacturer as potentially pathogenic bacteria not related to CE; observed in endometrial profiles by 16S sequencing (12, 13) | 0-451 | 80 | 0 (0.0) | 0 (0.0) | 0 (0.0) | - | - | - | - | - | - |
| Other reproductive-tract | Prevotella bivia | Classified by the manufacturer as potentially pathogenic bacteria not related to CE; observed in endometrial profiles by 16S sequencing (12, 13) | 0-603 | 1.6 | 4 (3.2) | 0 (0.0) | 4 (5.8) | 3,073 | 1,684-33,118 | 0.13 (0.01-2.40) | 0.126 | 1.000 | 1.000 |
| Other reproductive-tract | Prevotella disiens | Classified by the manufacturer as potentially pathogenic bacteria not related to CE; observed in endometrial profiles by 16S sequencing (12, 13) | 0-205 | 80 | 0 (0.0) | 0 (0.0) | 0 (0.0) | - | - | - | - | - | - |
| Other reproductive-tract | Sneathia spp. | Classified by the manufacturer as potentially pathogenic bacteria not related to CE; observed in endometrial profiles by 16S sequencing (12, 13) | 0-80 | 80 | 0 (0.0) | 0 (0.0) | 0 (0.0) | - | - | - | - | - | - |
| Other reproductive-tract | Treponema pallidum | Classified by the manufacturer as potentially pathogenic bacteria not related to CE; observed in endometrial profiles by 16S sequencing (12, 13) | Absent (any detection) | 160 | 0 (0.0) | 0 (0.0) | 0 (0.0) | - | - | - | - | - | - |
| Lactobacillus | Lactobacillus spp. (genus) | Taxon defining a Lactobacillus-dominant profile (12, 13) | n.a. | n.r. | 70 (55.6) | 27 (47.4) | 43 (62.3) | 2,394 | 166-355,000 | 0.54 (0.27-1.11) | 0.107 | n.a. | n.a. |

*The panel is a commercially pre-designed panel used in its entirety, with no organism added or omitted by the investigators. The manufacturer classifies the targets as Lactobacillus, bacteria most commonly associated with chronic endometritis, and other potentially pathogenic bacteria not related to chronic endometritis; the basis-for-inclusion column gives the literature basis for each group, as the manufacturer does not publish a target-by-target rationale. Results are genome copies per mL (GC/mL). Reference ranges were established by the manufacturer from the analysis of Moreno et al. (13). For targets designated "Absent", any detection is abnormal. Detection denotes amplification at a Crt of 28 or less (the platform detection cut-off) irrespective of the reference range; it was observed post hoc that among the 10 CE-associated targets the two definitions identified the same women, because every value reported already exceeded its reference limit, whereas Bifidobacterium spp. and Gardnerella vaginalis were detected below their reference limits in 14 and 5 women respectively. Fifteen targets were never detected. Odds ratios are cross-product ratios with a Haldane-Anscombe 0.5 correction where a cell was zero and Woolf 95% CIs; P values are from Fisher exact test. Benjamini-Hochberg and Holm adjustment was applied within each prespecified group (10 CE-associated targets; 16 other reproductive-tract targets), including targets never detected. Lactobacillus was one of the three prespecified category-level exposures rather than an exploratory target-level test and is therefore not included in either correction family (n.a.). In the broader family of all 26 non-Lactobacillus targets the adjusted P value for Escherichia coli was 0.446. No summed bacterial load was constructed. LOD, target-specific limit of detection published by the manufacturer for the laboratory serving Europe, Asia, Africa and Oceania, expressed as the lowest spike-in dilution point detected (genomic copies/µL) (29); for Streptococcus the values for group B (S. agalactiae) and S. viridans are listed separately by the manufacturer; n.r., not reported for the genus-level Lactobacillus assay. These values refer to spiked dilutions and are not directly comparable with the GC/mL values reported for clinical samples.*

**Supplementary Table S4. All sensitivity analyses for the association between each qPCR category and CD138 positivity.**

| **Analysis (n)** | **qPCR category** | **Detected, n** | **OR (95% CI)** | **P** |
| --- | --- | --- | --- | --- |
| Primary analysis (all women) (n = 126) | CE-associated pathogen targets | 13 | 8.01 (1.70–37.85) | 0.003 |
|  | Other reproductive-tract bacterial targets | 37 | 0.55 (0.25–1.22) | 0.171 |
|  | Lactobacillus | 70 | 0.54 (0.27–1.11) | 0.107 |
| Single + multiple polyps excluded (n = 118) | CE-associated pathogen targets | 12 | 17.44 (2.17–140.17) | <0.001 |
|  | Other reproductive-tract bacterial targets | 34 | 0.42 (0.18–0.98) | 0.065 |
|  | Lactobacillus | 67 | 0.53 (0.25–1.11) | 0.097 |
| All polyps excluded (incl. micropolyps) (n = 106) | CE-associated pathogen targets | 11 | 18.79 (2.30–153.21) | <0.001 |
|  | Other reproductive-tract bacterial targets | 30 | 0.43 (0.17–1.08) | 0.081 |
|  | Lactobacillus | 61 | 0.55 (0.25–1.21) | 0.163 |
| Endometriosis / adenomyosis excluded (n = 111) | CE-associated pathogen targets | 13 | 8.32 (1.75–39.60) | 0.003 |
|  | Other reproductive-tract bacterial targets | 32 | 0.65 (0.28–1.50) | 0.400 |
|  | Lactobacillus | 61 | 0.60 (0.28–1.28) | 0.250 |
| Endo/adeno + single/multiple polyps excluded (n = 103) | CE-associated pathogen targets | 12 | 18.44 (2.28–149.21) | <0.001 |
|  | Other reproductive-tract bacterial targets | 29 | 0.48 (0.19–1.18) | 0.125 |
|  | Lactobacillus | 58 | 0.58 (0.27–1.29) | 0.230 |
| Endo/adeno + all polyps excluded (n = 94) | CE-associated pathogen targets | 11 | 18.62 (2.27–152.76) | <0.001 |
|  | Other reproductive-tract bacterial targets | 25 | 0.45 (0.17–1.21) | 0.155 |
|  | Lactobacillus | 54 | 0.65 (0.28–1.49) | 0.398 |

*Odds ratios are cross-product ratios with logit (Woolf) 95% confidence intervals; P values are from Fisher exact test. Because the interval and the test are computed by different methods, a confidence interval that marginally excludes 1 may accompany an exact P value above 0.05 in small strata (for example OR 0.42, 95% CI 0.18–0.98, P = 0.065); inference was based on the exact test.*

**Supplementary Table S5. Lactobacillus analyses (N = 126).**

**S5a. Genus- and species-level detection**

| **Target** | LOD (genomic copies/µL) | **Detected, n (%)** | **CD138+ (n=57)** | **CD138- (n=69)** | **Median GC/mL** | **Range GC/mL** | **OR (95% CI)** | **P** |
| --- | --- | --- | --- | --- | --- | --- | --- | --- |
| Lactobacillus spp. (genus) | n.r. | 70 (55.6) | 27 | 43 | 2,394 | 166-355,000 | 0.54 (0.27-1.11) | 0.107 |
| L. crispatus | 8 | 24 (19.0) | 7 | 17 | 3,177 | 934-32,994 | 0.43 (0.16-1.12) | 0.11 |
| L. gasseri | 8 | 6 (4.8) | 1 | 5 | 2,945 | 932-10,446 | 0.23 (0.03-2.02) | 0.22 |
| L. iners | 80 | 22 (17.5) | 10 | 12 | 2,180 | 550-285,000 | 1.01 (0.40-2.55) | 1.0 |
| L. jensenii | 80 | 10 (7.9) | 6 | 4 | 2,477 | 410-69,474 | 1.91 (0.51-7.14) | 0.346 |

**S5b. CD138 positivity by stratum of reported Lactobacillus values**

| **Stratum** | **n** | **CD138-positive, n (%)** |
| --- | --- | --- |
| Undetected | 56 | 30 (53.6) |
| Below median of positives (<2,394 GC/mL) | 35 | 17 (48.6) |
| At or above median (>=2,394 GC/mL) | 35 | 10 (28.6) |

**S5c. Quantitative comparisons (genus)**

| **Analysis** | **CD138-positive** | **CD138-negative** | **P** |
| --- | --- | --- | --- |
| Lactobacillus load, all women including zero values, median (IQR) GC/mL | 0 (0-1,648) | 550 (0-4,312) | 0.045 |
| Lactobacillus load, Lactobacillus-positive women only (n=70) | 1,724 (1,088-3,076) | 3,726 (949-8,022) | 0.242 |
| Adjusted OR per 10-fold increase in Lactobacillus load (logistic) | 0.84 (95% CI 0.67-1.05) | - | 0.126 |

*No species was significantly associated with CD138 positivity; the point estimates nevertheless differed in direction between species (L. crispatus 0.43, L. iners 1.01, L. jensenii 1.91), and genus-level analysis combines these species into a single variable and cannot detect such differences (Supplementary Figure S3). The unadjusted comparison of reported Lactobacillus values including women with no detectable Lactobacillus gave P = 0.045; the higher proportion of women with no detectable Lactobacillus in the CD138-positive group contributed to this difference, and the comparison restricted to the 70 Lactobacillus-positive women was not significant (P = 0.242). This exploratory result was not corrected for multiplicity. Values below the detection threshold are reported as zero but are not necessarily true zeros. The three strata use the median among Lactobacillus-positive samples (2,394 GC/mL) as the cut-off, which is data-derived; the comparison across the three strata was not significant (chi-square P = 0.059) and the adjusted odds ratio per 10-fold increase in the reported value was 0.84 (95% CI 0.67-1.05, P = 0.126). Taken together these analyses are directionally consistent but not confirmatory. Lactobacillus dominance in the sense used in sequencing studies could not be assessed. LOD, limit of detection published by the manufacturer, as in Supplementary Table S3 (29); n.r., not reported.*

**Supplementary Table S6. CD138 positivity by the result of the manufacturer’s decision tree for Lactobacillus and the other reproductive-tract bacterial targets (N = 126).**

| Manufacturer decision-tree result | **n** | **CD138-positive, n (%)** | **OR (95% CI)** | **P** |
| --- | --- | --- | --- | --- |
| Optimal | 63 | 26 (41.3) | 1.00 (reference) | - |
| Non-optimal: other reproductive-tract target out of reference range | 24 | 7 (29.2) | 0.59 (0.21-1.61) | 0.334 |
| Non-optimal: Lactobacillus not detected | 39 | 24 (61.5) | 2.28 (1.01-5.16) | 0.066 |

*Groups follow the decision tree of the current manufacturer’s manual (Manual EndomeTRIO Custom OA, version 3.1, 2025): optimal, no other reproductive-tract target out of its reference range and Lactobacillus detected; non-optimal, at least one other reproductive-tract target out of its reference range; non-optimal, all such targets within range but Lactobacillus not detected. The reference ranges were calculated by the manufacturer from samples of women with a live birth in the study of Moreno et al. (13). CE-associated pathogen targets are evaluated in a separate manufacturer decision tree (out of reference range, treatment indicated), which identifies the same 13 women as the primary exposure and is therefore not repeated here. Comparison across the three groups: chi-square P = 0.029. The non-optimal result driven by out-of-range targets was not associated with CD138 positivity; the association ran through the group in which Lactobacillus was not detected. The classification is a per-target threshold rule and does not require summing copy numbers across assays, so it is the closest available community-level readout in this dataset; it uses the reference range rather than the detection threshold used for the three prespecified categories, which is why 24 women are classified as non-optimal because of out-of-range targets whereas 37 had at least one other reproductive-tract target detected. As in Supplementary Table S4, the Woolf interval and the exact test may disagree marginally (OR 2.28, 95% CI 1.01–5.16, P = 0.066).*
